# Minimally symptomatic cerebral amyloid angiopathy-related inflammation: three descriptive case reports

**DOI:** 10.1136/jnnp-2017-317347

**Published:** 2018-03-13

**Authors:** Gargi Banerjee, Debie Alvares, John Bowen, Matthew E Adams, David J Werring

**Affiliations:** 1 Stroke Research Centre, Department of Brain Repair and Rehabilitation, UCL Institute of Neurology and the National Hospital for Neurology and Neurosurgery, London, UK; 2 Department of Neurology, St. Richard’s Hospital, Western Sussex Hospitals NHS Foundation Trust, Chichester, UK; 3 Department of Neurology, Royal Shrewsbury Hospital, The Shrewsbury and Telford Hospital NHS Trust, Shrewsbury, UK; 4 Lysholm Department of Neuroradiology, National Hospital for Neurology and Neurosurgery, London, UK

**Keywords:** amyloid, cerebrovascular disease, clinical neurology, mri, neuroradiology

## Introduction

Cerebral amyloid angiopathy-related inflammation (CAA-ri) is an unusual cause of encephalopathy, seizures and focal neurological deficits.^1 2^ We report three cases of CAA-ri with minimal symptoms but striking and dynamically evolving brain MRI findings.

## Case 1

A 62-year-old man presented with a moderately severe non-radiating frontal headache. Brain MRI 9 months later showed multiple discrete regions of abnormal signal and mild swelling involving white matter and overlying cortex. Susceptibility-weighted imaging (SWI) demonstrated numerous cortical lobar microbleeds throughout both cerebral hemispheres. Repeat MRI another 9 months later showed resolution of many of the parenchymal abnormalities, but with several new regions containing more peripheral microbleeds. Amyloid-PET (using ^18^F-florbetapir) demonstrated moderate widespread amyloid deposition; CSF analysis showed reduced amyloid-beta 1–42 and high-normal total tau. Formal neuropsychological testing suggested mild compromise in frontal functioning only. The patient was treated with 5 days of intravenous methylprednisolone (1 g daily), followed by an oral taper from prednisolone 60 mg over 8 weeks. Follow-up MRI after 8 months showed almost complete resolution of the parenchymal abnormalities, but with persisting lobar microbleeds. At 24 months following symptom onset, he remains asymptomatic, with stable brain imaging.

## Case 2

A 74-year-old man presented with mild subjective memory difficulties only, with no objective neuropsychological deficits. MRI demonstrated a substantial region of abnormal signal in the right temporal and occipital white matter, with no enhancement. Repeat imaging after a few weeks showed partial regression. Over the following 4 years, three further MRIs showed multiple areas of abnormal white matter (sometimes involving cortex as well) within the temporal, parietal and occipital lobes, which largely resolved. SWI demonstrated progressive accumulation of lobar microbleeds, mainly in the affected areas. The patient remains asymptomatic with no change in his subjective cognitive symptoms, without having received immunosuppressive treatment.

## Case 3

**Figure 1:**
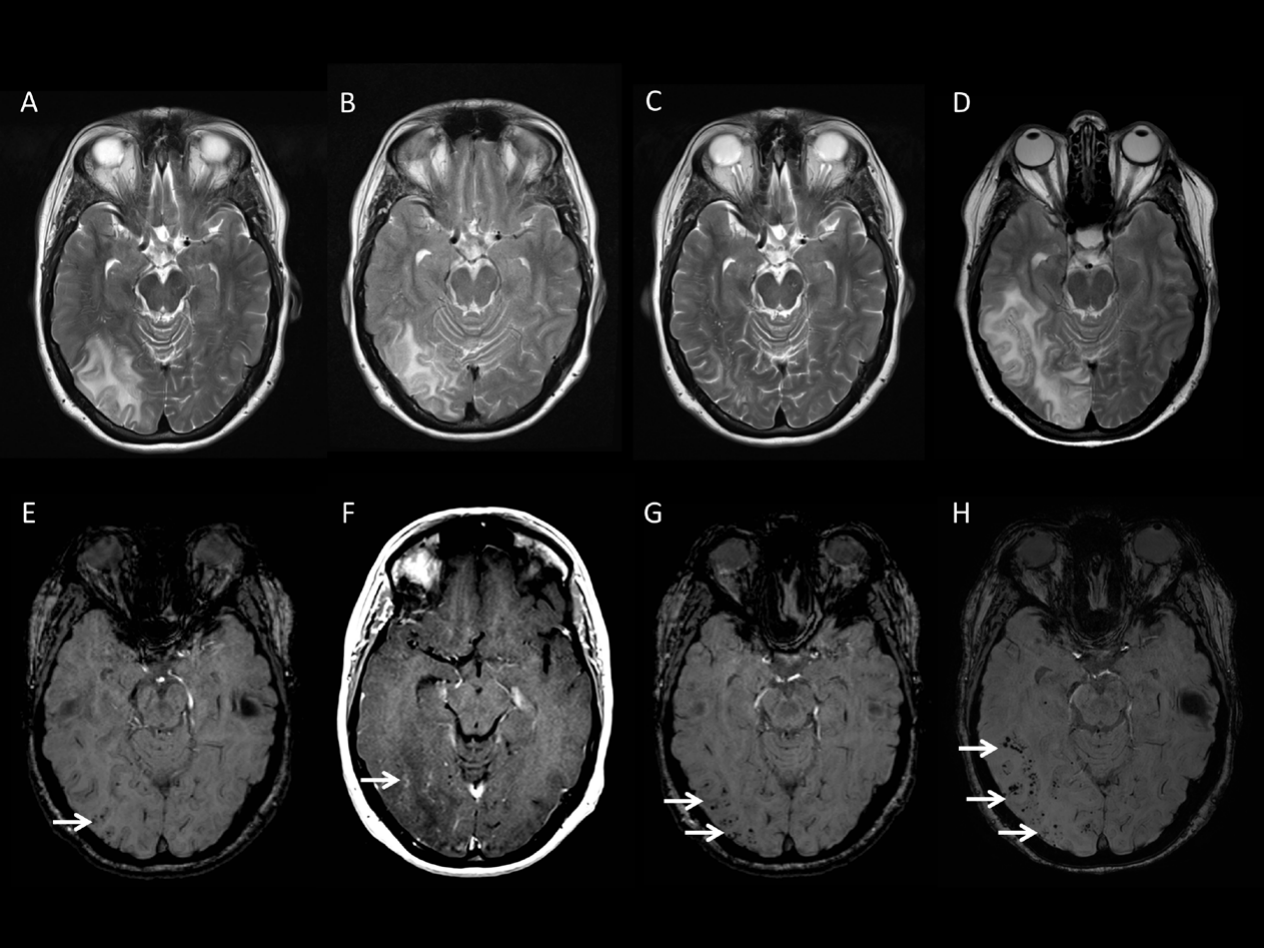
MRI from Case 3, illustrating the incidence of different imaging features of CAA-ri. T2-weighted images obtained 9 months following intitial presentation (A) demonstrate an area of parenchymal signal abnormality in the right temporo-occipital region. SWI from the same time (E) show a few cortical microbleeds. Further imaging obtained 2 months later shows progression of the right temporo-occipital abnormalities on T2-weighted sequences (B) and post-gadolinium T1-weighted images (G) show only subtle enhancement (F). The patient was then treated with corticosteroids, and T2-weighted MRI 5 weeks later shows significant improvement of the abnormalities (C), while SWI demonstrates an increase in the number of cortical microbleeds in the affected area (G). The patient developed new visual symptoms 1 year following her corticosteroid treatment and was reimaged. T2-weighted imaging (D) showed recurrence and extension of the original right temporo-occipital parenchymal abnormalities, with the coincident development of multiple new cortical microbleeds (H). CAA-ri, cerebral amyloid angiopathy-related inflammation; SWI, susceptibility weighted images.

A 54-year-old woman presented with a bright flashing light in her left visual field and a sudden onset headache. After initial CT of the brain demonstrated right-sided occipital hypoattenuation, she was treated for ischaemic stroke and then antiepileptic drugs for presumed seizures. Approximately 6 months later, she developed worsening headache; MRI showed an area of abnormal signal and mild parenchymal swelling in the right temporo-occipital area. A diagnostic brain biopsy showed CAA-ri (Vonsattel grade 3 CAA with associated chronic inflammatory cell infiltration within and around the vessel wall, with angiodestructive and occlusive features). After a further 8 months, she was still experiencing occasional left-sided visual flickering and some subtle memory difficulties. MRI (figure 1) demonstrated progression of the right temporo-occipital abnormality, together with a new separate focus in the anterior right temporal lobe and multiple lobar microbleeds in these regions. Formal neuropsychological testing was normal. Although clinically stable, further MRI 7 weeks later showed extension of the right temporal lobe lesion. She was treated with intravenous methylprednisolone (1 g daily, 5 days, followed by tapering dose prednisolone); 1 month later, the parenchymal signal abnormalities had improved significantly, with no increase in the number of microbleeds.

One year after intravenous corticosteroid treatment, while still taking oral steroids, the patient developed headache and new left-sided visual disturbances. MRI showed recurrence and extension of the right-sided temporo-occipital region abnormalities, with local swelling and numerous new cortical microbleeds in the affected area. The patient was once again treated with intravenous corticosteroids (as previously); follow-up MRI 3 months after this showed almost complete regression of the right temporal abnormalities and no change in the appearance or number of peripheral microbleeds.

## Discussion

We report three cases of CAA-ri (one definite and two probable, according to proposed criteria for CAA-ri^1 2^) in which the diagnosis of CAA-ri was made when the patients underwent neuroimaging for mild neurological symptoms. Imaging in all cases showed regions of abnormal gyral signal that waxed and waned over time (months to several years) and involved multiple separate areas, either simultaneously or sequentially, often in the absence of new clinical features. Peripheral lobar microbleeds were observed in all cases and tended to accumulate in areas affected by the abnormal MRI signal and swelling. While headache and positive visual symptoms have been described in CAA-ri, these occurred with more serious neurological symptoms (coma, seizures, altered behaviour, focal neurological deficits^3^); to the best of our knowledge, CAA-ri presenting with minimal or no symptoms has not previously been described.

These cases highlight several points of interest. The first is the dissociation between the mild clinical features and striking radiological abnormalities; this has been described during the routine follow-up in patients with known CAA-ri.^4^ Greater MRI availability and an increasing awareness of CAA-ri might thus result in more incidentally diagnosed cases. A recent case series^5^ described three patients presenting with acute stroke (one ischaemic, two haemorrhagic) with coexistent MRI and cerebrospinal fluid evidence of CAA-ri.

Consistent with previous reports,^6–8^ our cases closely resemble the amyloid-related imaging abnormalities (ARIA) described in patients with Alzheimer’s disease treated with antiamyloid immunotherapy.^9^ However, CAA-ri is usually associated with marked neurological disturbances, while ARIA is often asymptomatic or mild.^10^ Imaging findings consistent with CAA-ri or ARIA have also been described in a small number of patients with Alzheimer’s disease prior to treatment, all of whom were asymptomatic.^11^ Our cases suggest that spontaneous CAA-ri can also present with minimal symptoms and so might represent a normal physiological mechanism of amyloid clearance.^12^

Pathological verification remains the gold standard for CAA-ri (only available for one of our patients) and the current clinico-radiological criteria require further validation, particularly for atypical cases, where amyloid- Positron Emission Tomography (PET)^13^ and cerebrospinal fluid (CSF) findings^14^ might be helpful. Additionally, minimally symptomatic cases might differ from ‘classical’ CAA-ri; for example, the ApoE ε4 allele is associated with CAA-ri;^14^ but we did not obtain ApoE information and so cannot investigate this. Although our case reports expand the clinical spectrum of CAA, further longer-term follow-up to better establish the natural history of minimally symptomatic CAA-ri is needed.
